# Transcriptome analysis of *Rhizopus oryzae* seed pellet formation using triethanolamine

**DOI:** 10.1186/s13068-021-02081-y

**Published:** 2021-12-04

**Authors:** Na Wu, Jiahui Zhang, Wen Ou, Yaru Chen, Ru Wang, Ke Li, Xiao-man Sun, Yingfeng Li, Qing Xu, He Huang

**Affiliations:** School of Food Science and Pharmaceutical Engineering, Nanjing Normal University, Nanjing, China

**Keywords:** *R. oryzae*, Seed pellet formation, Triethanolamine, Organic acid, Transcriptomic analysis

## Abstract

*Rhizopus oryzae* (*R. oryzae*) can effectively produce organic acids, and its pellet formation in seed cultures has been shown to significantly enhance subsequent fermentation processes. Despite advances in strain development, simple and effective methods for inducing pellet morphology and a basic understanding of the mechanisms controlling this process could facilitate substantial increases in efficiency and product output. Here, we report that 1.5% triethanolamine (TEOA) in seed culture medium can activate the growth of *R. oryzae* spores in compact and uniform pellets which is optimal for fermentation conditions. Analysis of fermentation kinetics showed that the production of fumaric and L-malic acid increases 293% and 177%, respectively. Transcriptomic analysis revealed that exposure of *R. oryzae* to 1.5% TEOA during the seed culture activated the phosphatidylinositol and mitogen-activated protein kinase signaling pathways. Theses pathways subsequently stimulated the downstream carbohydrate-active synthases and hydrolases that required for cell wall component synthesis and reconstruction. Our results thus provide insight into the regulatory pathways controlling pellet morphology germane to the viability of seed cultures, and provide valuable reference data for subsequent optimization of organic acid fermentation by *R. oryzae*.

**Supplementary Information:**

The online version contains supplementary material available at 10.1186/s13068-021-02081-y.

## Introduction

Depletion of fossil fuel resources combined with excessive CO_2_ emissions has resulted in a growing environmental and energy crisis. The field of biorefinery aims to use microorganisms to convert renewable biomass into chemicals, fuels, and materials, offering a promising route to address these challenges [1, 2]. Among the microorganisms used in biorefinery processes, filamentous fungi occupy a uniquely favorable position due to their characteristically rapid propagation, high biomass density, and broad substrate range [3, 4]. Filamentous fungi can thus serve as a viable platform to fabricate valuable products, such as enzymes, organic acids, vitamins, amino acids, antibiotics, and fatty acids [5, 6]. However, filamentous fungi frequently exhibit diverse morphologies, including clumps, filaments, or pellets, which are accompanied by differences in oxygen diffusion and mass transfer capacity, greatly affecting the fermentation performance [7]. Previous studies have confirmed that pellet morphology can increase the productivity of submerged fermentation processes, since pellets can significantly lower the viscosity of the medium compared to filamentous hyphae, thus facilitating the uptake of substrates and oxygen [8]. Therefore, reliable methods for facilitating pellet formation and optimizing pellet structure during the seed culture of filamentous fungi have become a widely sought goal in the fermentation community [9].

Several strategies have been proposed to achieve this goal, including adjusting the medium composition (e.g*.,* carbon source, nitrogen source, metal ions), supplementing the medium with additives (polymers, surfactants, or chelators), and changing the culture conditions (density of spore inoculum, temperature, pH, or agitation speed) [10–14]. For example, Iyyappan et al*.* showed that careful adjustment of the composition of the seed medium, spore inoculum concentration, and shaking speed can help optimize the pellet morphology of *Aspergillus niger* for the production of L-malic acid from glycerol [15]. Similarly, Kurakake et al. found that the mycelial pellets of *Aspergillus oryzae* became smaller and spherical following the addition of nonionic surfactants, resulting in enhanced production of *β*-fructofuranosidase [16]. Similarly, Gao et al*.* demonstrated that the morphology of the oleaginous fungus *Mortierella isabellina* could be precisely controlled by adding magnesium silicate microparticles [17].

Among filamentous fungi domesticated for biorefinery processes, *Rhizopus oryzae* (*R. oryzae*) exhibits a distinct and remarkable capacity for the production of fumaric acid, L-malic acid, and lactic acid [2]. Previous research has shown that organic acid yields after submerged fermentation are correlated with seed morphology, leading to subsequent efforts to promote pellet formation and determine the optimal pellet structure [9]. For example, Zhou et al. manipulated the pellet morphology by adding soybean peptone and inorganic ions, thereby improving fumaric acid production by 46.13% [18]. Das et al. studied the effects of pH, temperature, shaking speed, and inoculum concentration on the morphology of *R. oryzae* 1526 and reported that higher fumaric acid production could be obtained by inducing pellet formation [19]. However, the underlying regulatory mechanisms controlling the induction of pellets are still unknown and straightforward protocols for activating these mechanisms are lacking.

**Fig. 1:**
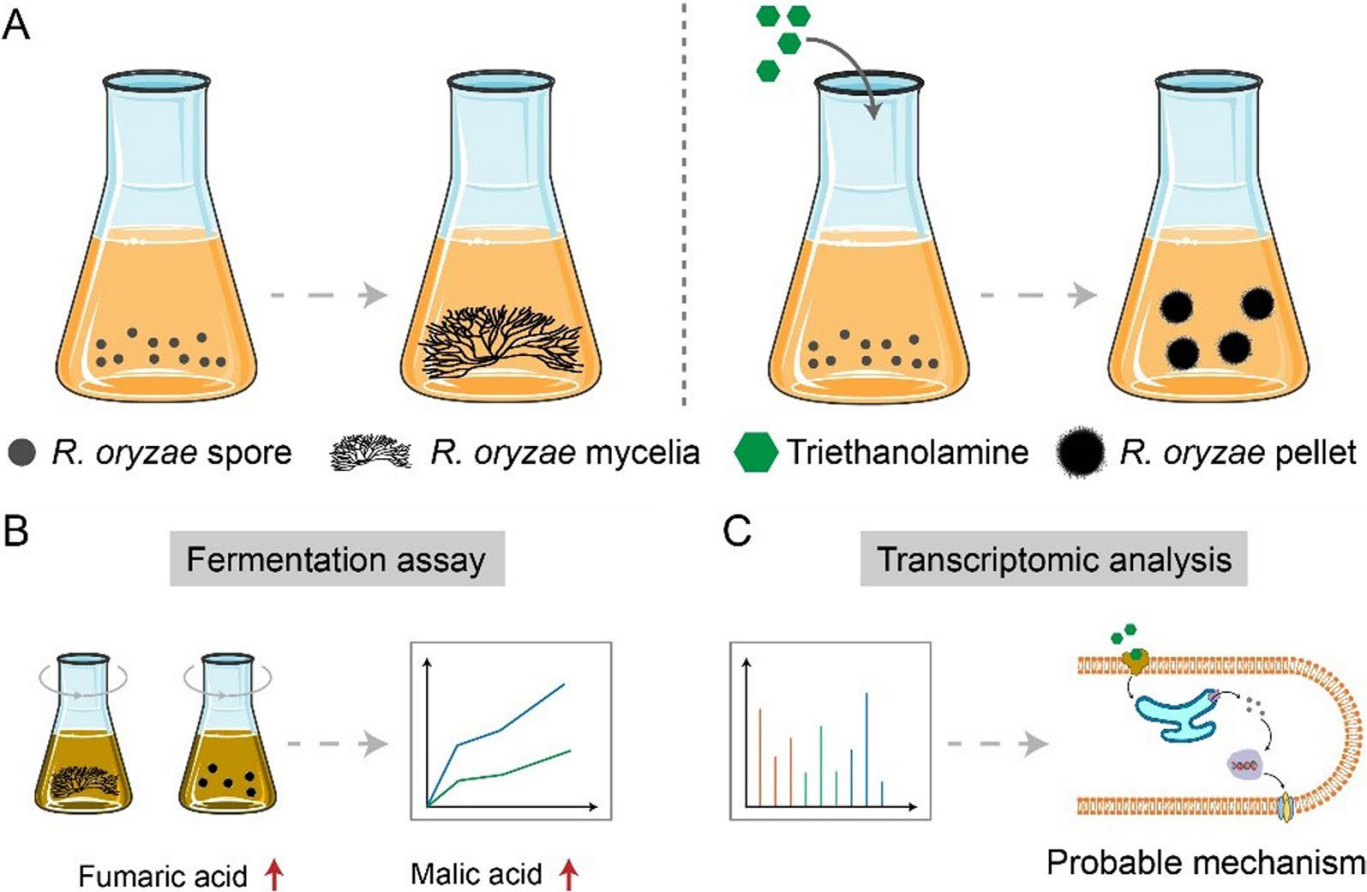
Scheme of the experimental strategy and analysis of *R. oryzae* pellet formation induced by surfactants. **A** Illustration of *R. oryzae* seed pellet formation induced by triethanolamine (TEOA). **B** Fermentation assay of *R. oryzae* with different seed morphologies. **C** Transcriptomic analysis revealing the probable mechanism of pellet formation

Here, we reveal that supplementation with the surfactant triethanolamine (TEOA) can promote *R. oryzae* pellet formation in fermentation seed cultures (Fig. 1). We first screened several surfactants and found that TEOA was the strongest inducer of pellet morphology. We next investigated the effects of different concentrations of TEOA on *R. oryzae* pellet size at the end of the seed culture and tested the corresponding organic acid outputs after fermentation (Fig. 1B). The results showed that supplementing the seed culture with 1.5% TEOA resulted in the highest production of fumaric and L-malic acid. Transcriptomic analysis revealed that TEOA activates carbohydrate-active enzymes to synthesize and restructure the cell wall via several critical signaling pathways (Fig. 1C). Our findings can thus provide insight into the basic mechanisms underlying pellet morphology in response to TEOA, and can thus be used, in conjunction with our reference data for glucose substrate, as a framework to guide improvements in fermentative production of organic acids by *R. oryzae*.

## Materials and methods

### Strains, media and growth conditions

*R. oryzae* ATCC20344 was purchased from the Microbial Species Preservation Center (Guangdong, China), and was first grown on potato-dextrose agar (PDA) at 35 ℃ for 5 days. Then, the spores of *R. oryzae* were washed with sterilized water. The spore suspension was carefully drawn by serological pipette, and adjusted with sterile water to concentrations of 1 × 10^7^ spores/mL using a hemocytometer (Sigma-Aldrich, China). Then, 1 mL of the seed suspension (1 × 10^7^ spores/mL) was used to inoculate 50 mL of seed culture medium (30 g/L glucose, 0.5 g/L MgSO_4_·7H_2_O, 0.6 g/L KH_2_PO_4_, 0.0088 g/L FeSO_4_·7H_2_O, 2 g/L urea and 0.11 g/L ZnSO_4_·7H_2_O) and grown at 35 ℃ and 200 rpm for 30 h. In order to induce pellet formation, different kind of different concentrations of triethanolamine (0, 0.5, 1.0, 1.5, 2.0%, V/V) were added to the seed culture medium.

Subsequently, 10% of the seed culture was transferred into 50 mL of fermentation medium (same as the seed culture medium but with 60 g/L glucose, 0.1 g/L urea, and 40 g/L CaCO_3_) and cultured at 35 ℃ and 200 rpm for 72 h.

### Morphological observation of *R. oryzae*

According to a previously reported method [9], we conducted morphological observations of *R. oryzae* using field emission scanning electron microscopy (S-4800, Hitachi, Japan). Briefly, samples were fixed with 2.5% glutaraldehyde and washed with PBS 3 times. Then, the samples were dehydrated in a series of ethanol solutions with a concentration of 30, 50, 70, 80, 90, 95, and 100% 15 min each. Finally, the samples were freeze-dried to observe the hyphal morphology.

### Measurement of organic acids, glucose and biomass

Organic acids were quantified by high-performance liquid chromatography (HPLC). A sample comprising 2 ml of the fermentation broth was added into 10 mL EP tubes, and an equal volume of 2 M HCl was added to fully acidify the sample. Then, the mixture was centrifuged at 12,000×*g* for 10 min, and the supernatant was filtered through a 0.22-μm pore-size filter membrane for HPLC analysis on an Aminex HPX-87H column (BioRad, USA) and a UV detector at 210 nm. The mobile phase consisted of 5 mM H_2_SO_4_ at a flow rate of 0.6 mL/min and the column temperature was 30 ℃. All measurements were carried out in three replicates.

Glucose was detected using an SBA-40C dual channel biosensor analyzer (Jinan Yanhe Biotechnology Company, China). Biomass was determined by washing the mycelia with water and drying at 70 ℃ to a constant weight.

### RNA sequencing and data analysis

*Rhizopus oryzae* was incubated in seed culture medium (SCM) containing 1.5% triethanolamine and control medium (SCM_Con) at 35 ℃ and 200 rpm for 24 h. Samples were collected by centrifugation at 5000×*g* for 10 min, and the pellets were immediately frozen in liquid nitrogen for RNA extraction using TRIzol reagent (Invitrogen, USA). In order to remove genomic cDNA, the extracted total RNA was incubated with DNase I (Takara, China) at 37 °C for 30 min. The cDNA library was constructed using the TruSeq™ RNA sample prep kit (Illumina, CA, USA). The mRNA-seq libraries were constructed using an Illumina NovaSeq 6000platform provided by Major Bio Co., Ltd, (Shanghai, China). The raw reads were processed by removing reads with adapters, poly-N and low-quality reads; SeqPrep (https://github.com/jstjohn/SeqPrep) was then used to clean the raw reads. The clean reads were then assembled using Trinity and searched against the public database Kyoto Encyclopedia of Genes and Genomes (KEGG).

Differentially expressed genes (DEGs) between the two groups (*R. oryzae* incubated in seed culture medium with 0 vs. 1.5% triethanolamine) were identified using the DESeq2 package using an adjusted-p value < 0.05 and |log2Fold change|> 1 as the screening criteria. Each experiment was carried out in three biological replicates.

## Results and discussion

### Effects of different surfactants on the seed morphology of *R. oryzae*

We first chose six surfactants, Triton 100, Tween 80, Tween 20, diethanolamine, ethanolamine, and TEOA as candidate additives to assess their respective effects on *R. oryzae* morphology in seed medium. These candidate surfactants were selected based on prior investigations showing that they could modulate the morphology of several different filamentous fungi [16, 20–23]. In order to thoroughly analyze the molecular mechanism regarding to seed pellet formation, we chose the unmutagenized *R. oryzae* strain as a test object. Digital images showing the colony morphology after 30 h of culture in seed medium with different surfactants (1.5% V/V) showed a range of responses by *R. oryzae* (Additional file 1: Fig. S1). However, we found that pellet formation only occurred with the addition of TEOA, whereas Triton 100, Tween 80, and Tween 20 had no discernible effect, with cultures consisting of suspended mycelia similar to untreated controls (Additional file 1: Fig. S1). Additionally, spores failed to germinate in medium containing diethanolamine or ethanolamine, leading us to speculate that diethanolamine and ethanolamine were not biocompatible with this species and likely inhibited hyphal growth (Additional file 1: Fig. S1).

We observed substantial differences in morphological features between the surfactant-free control group and the TEOA-supplemented group. In particular, the control group grew as intertwined, flocculent hyphae, while TEOA treatment resulted in the formation of uniformly dispersed pellets (Additional file 1: Fig. S1). In light of previous studies showing that aggregation into fungal pellets is essential for efficient production of organic acids, we concluded that TEOA was the only viable option among the candidate surfactants for further optimization of pellet induction to improve bioreactor fermentation of organic acids.

### Effects of different TEOA concentrations on *R. oryzae* seed

**Fig. 2:**
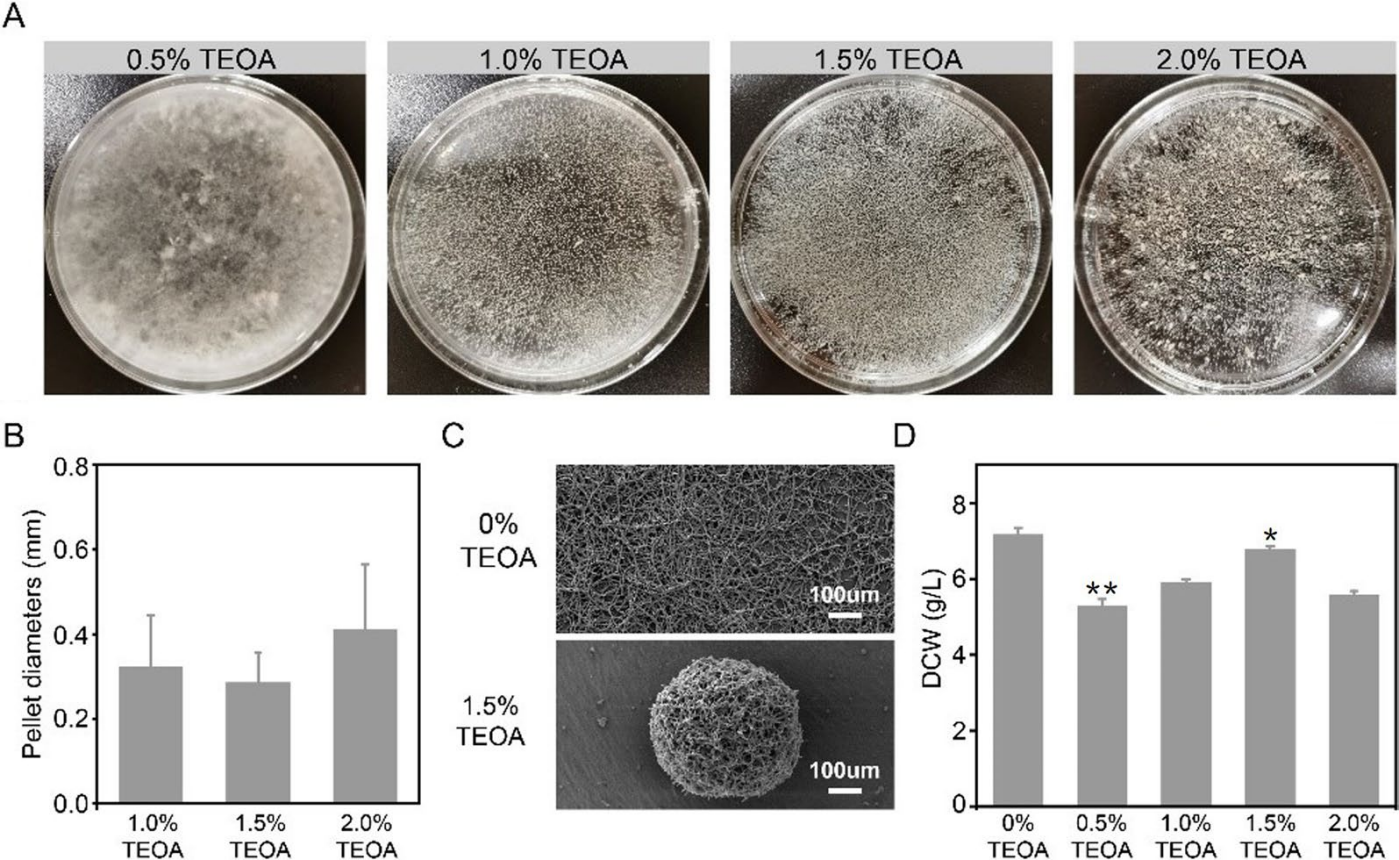
Optimization of TEOA addition in seed culture medium. **A** Different *R. oryzae* seed morphologies induced by different concentration of TEOA. **B** Effects of the triethanolamine concentration on *R. oryzae* seed pellet diameter. **C** SEM images of *R. oryzae* after seed culture with or without 1.5% TEOA. **D** Effects of the triethanolamine concentration on the CDW of *R. oryzae* seed cultures. Data represent means ± SD of three independent replicates. Statistical significance was determined by Student’s t test (n = 3). *p < 0.05, **p < 0.01

We next systematically investigated the relationship between the TEOA concentration in seed culture medium and the *R. oryzae* seed. To this end, we examined a range of TEOA concentrations (0.5, 1.0 1.5, and 2.0% v/v), and found that under 0.5% TEOA, the majority of hyphae were flocculent (Fig. 2A, left), indicating that this concentration was insufficient to induce the pellet morphology of *R. oryzae*. By contrast, we observed uniform pellet formation at TEOA concentrations of 1.0 and 1.5% (Fig. 2A, center), while further increase to 2.0% resulted in larger, but heterogeneous pellets (Fig. 2A, right). These results suggested that the TEOA concentrations could be adjusted to maximize the size and uniformity of *R. oryzae* pellets, with excessive TEOA leading to larger, but less predictable pellets.

We also quantified the pellet diameter and biomass in seed cultures of *R. oryzae* grown with different concentrations of TEOA*.* We assessed about 400 pellets and found relatively little change in the average diameter between 1.0 and 1.5% TEOA (0.32 ± 0.12 mm vs. 0.29 ± 0.07 mm), while the diameter at 2% TEOA (0.41 ± 0.15 mm) was obviously greater than at lower concentrations (Fig. 2B). We next used scanning electron microscopy (SEM) to further explore the differences of ultrastructure between the hyphae grown with and without 1.5% TEOA. The SEM images indicated that the surfactant-free *R. oryzae* grew as dispersed and branched mycelia, while the 1.5%TEOA group showed typical pellet morphology, highlighting the effectiveness of inducing seed pellet formation using TEOA (Fig. 2C).

The cell dry weight (CDW) of the surfactant-free *R. oryzae* culture was 7.17 ± 0.15 g/L, and significantly more in the TEOA addition groups (Fig. 2D). We proposed two possible explanations for this phenomenon. First, *R. oryzae* spores growing into compact pellet morphology could be subjected to physical and spatial constraints, leading to a decrease of the total biomass. Alternatively, the relative biocompatibility of TEOA as a surfactant may not preclude potentially adverse effects on fungal growth. The biomass increased with the addition of TEOA from 1.0 to 1.5%, reaching a maximum of 6.78 ± 0.08 g/L, but decreased to 5.58 ± 0.10 g/L under exposure to 2.0% TEOA (Fig. 2D). In order to further analyze the toxicity influence of TEOA on *R. oryzae*, we added the morphological observation experiments. We cultured *R. oryzae* spores on the PDA solid medium supplemented with different concentrations (1.5% and 2.0%) of TEOA. After 16 h, we observed that the colony diameter of the 1.5% TEOA-supplemented group was 0.51 ± 0.04 cm (Additional file 1: Fig. S2A and 2B). However, the diameter of 2% TEOA-supplemented group was significantly smaller (0.33 ± 0.05 cm), implying that TEOA at 2% concentration have more toxicity that 1.5% concentration (Additional file 1: Fig. S2A and 2B). Therefore, we speculated that the high concentration (2%) of triethanolamine may induce toxicity and inhibit of *R. oryzae* growth. Previous studies have demonstrated that TEOA could inhibit the proliferation of human muscle fibroblasts cells [24]. Our subsequent transcriptome analysis showed that growth on 1.5% TEOA-supplemented medium during the seed culture process induced a stress response in *R. oryzae*. Notably, our finding that seed cultures with the most compact pellets (i.e., 1.5% TEOA) also had the highest CDW was in agreement with previous studies showing that fungal biomass tended to increase with decreasing pellet diameter [18]. These results implied that hyphal growth was restrained under conditions promoting the formation of larger pellets.

### Fermentation kinetics of *R. oryzae* with different seed morphologies

**Fig. 3:**
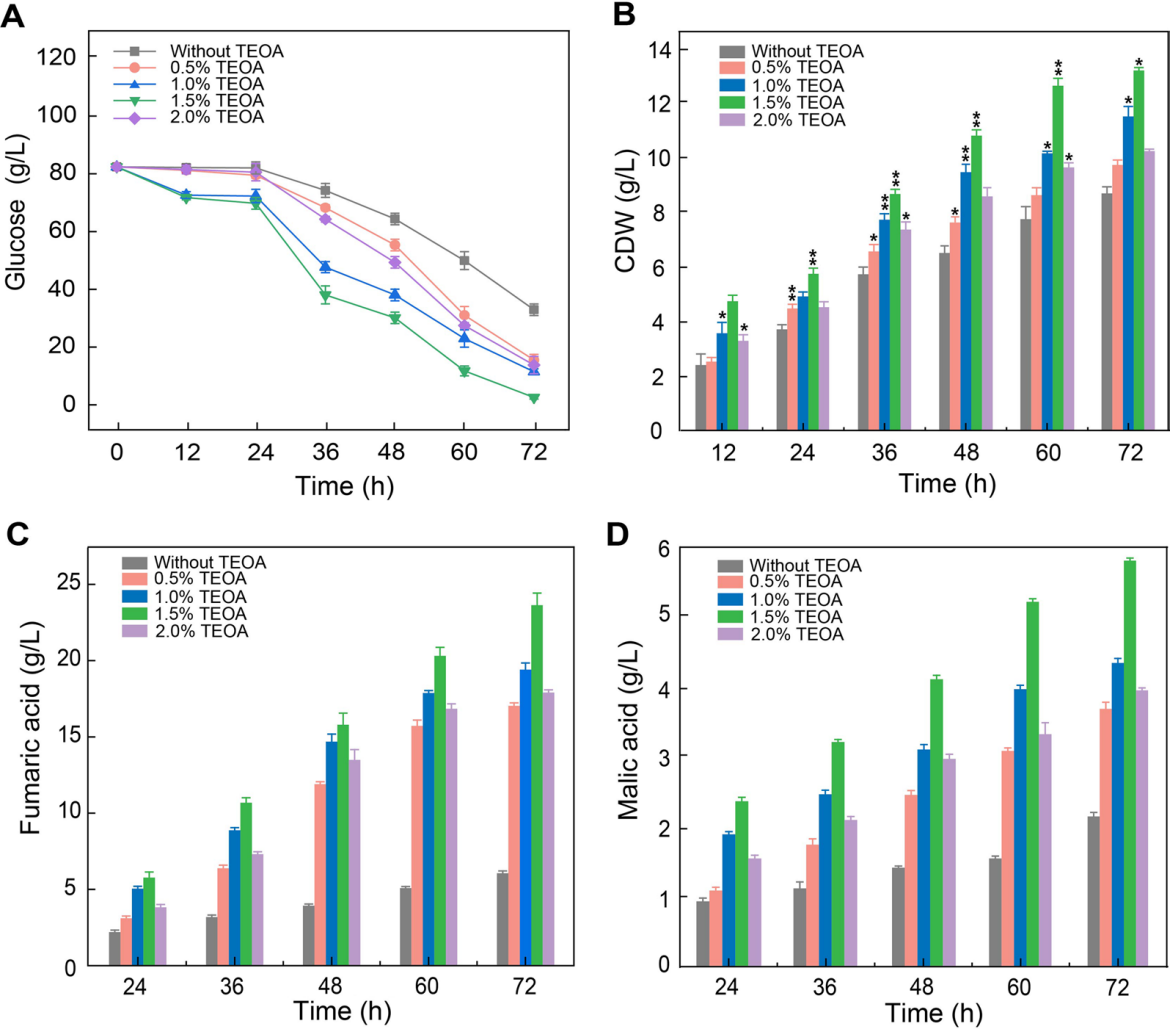
Fermentation kinetics of *R. oryzae* with different morphologies formed by adding different dosages of TEOA in seed culture medium. **A** Glucose concentration in fermentation medium. **B** CDW. **C** Fumaric acid production. **D** Malic acid production. Data represent means ± SD of three independent replicates. Statistical significance was determined by Student’s t test (n = 3). * p < 0.05, ** p < 0.01

We next tested the fermentation parameters of *R. oryzae* seed cultures with different morphologies induced by different concentrations of TEOA (0, 0.5, 1, 1.5, 2.0%). To exclude the influence of TEOA during the fermentation process, we first collected the seeds through centrifugation and washed them with deionized water three times. Then, we transferred the seeds into fermentation medium. We focused on several parameters, including glucose consumption, CDW, fumaric acid production, and L-malic acid production. Glucose consumption was increased in all groups after 24 h, and the glucose consumption of the surfactant-free group was significantly lower than that of the TEOA-treated groups after 72 h (Fig. 3A). The 1.5%TEOA group consumed almost all the glucose, while the other TEOA-treated groups (0.5, 1, 2%) had a residual glucose concentration of about 10 g/L (Fig. 3A). By contrast, the surfactant-free group had a residual glucose concentration of 30.64 ± 0.10 g/L (Fig. 3A). Previous studies have shown limitations in diffusive mass transfer in larger diameter cell pellets of filamentous fungi, thus reducing accessibility to nutrients and oxygen, especially at the center of the pellet. In addition, mycelial clumps can also lead to these same difficulties with mixing and limited diffusion of oxygen and nutrients, which reduces microbial growth and metabolism [25]. The smaller pellets could improve the oxygen transfer to the fungal cells [26, 27]. The TEOA-treated group was amenable to pellet formation, and at the optimal TEOA concentration, *R. oryzae* forms smaller, more uniform pellets. Therefore, inducing a smaller pellet size by TEOA treatment could ultimately improve oxygen transfer to individual cells in the pellet, enhancing subsequent growth and metabolism, and ultimately accelerating glucose consumption following inoculation of fresh fermentations.

Similarly, the 1.5% TEOA group exhibited the largest CDW (13.07 ± 0.10 g/L) after 72 h of fermentation, while the surfactant-free group had the smallest CDW of 8.60 ± 0.24 g/L (Fig. 3B). We speculated that although the surfactant-free group had the largest biomass after the seed culture process, the branched morphology reduced the mass transfer rate of glucose and oxygen, resulting in a decrease of the final CDW [18]. In addition, we inferred that the largest CDW of the 1.5% TEOA group may be attributed to its largest seed biomass after the seed culture process. We next focused on the crucial fermentation parameter, organic acid production.

Fumaric acid (trans-1,2-ethylene dicarboxylic acid, C_4_H_4_O_4_) is an essential platform chemical, and it is currently mainly synthesized from petrochemicals [28]. Previous investigations revealed that fumaric acid is the prominent metabolite of *R. oryzae* fermentation [29–32]. In our fermentation kinetics assays, we observed that the highest titer of fumaric acid obtained during fermentation was produced by the 1.5% TEOA group, reaching 23.64 ± 0.80 g/L after 72 h, with a yield of ∼ 0.30 g/g (fumaric acid/glucose) and a productivity of ∼ 0.33 g/L/h. These values were 293%, 150%, and 313% higher than those of the surfactant-free group (6.02 ± 0.15 g/L, ∼ 0.12 g/g, ∼ 0.08 g/L/h), respectively (Fig. 3C). Moreover, the titers, yields and productivity of all the TEOA-treated groups were significantly greater than in the untreated group after 72 h of fermentation, confirming the effectiveness of our strategy (Fig. 3C).

L-malic acid, also referred to as (S)-2-hydroxysuccinic acid (C_4_H_6_O_5_), is recognized as a valuable specialty chemical with broad applications in the food and pharmaceutical industries [33]. As an intermediate of the tricarboxylic acid (TCA) cycle, L-malic acid is also accumulated during *R. oryzae* fermentation [34]. In our investigation, we also found that the 1.5% TEOA group exhibited the highest titer, yield, and productivity of L-malic acid (5.77 ± 0.80 g/L, ∼ 0.07 g/g, ∼ 0.08 g/L/h), whereas the surfactant-free group had a titer of 2.13 ± 0.04 g/L, a yield of ∼ 0.04 g/g, and a productivity of ∼ 0.03 g/L/h in 72 h (Fig. 3D). Moreover, we found that the 1.5% TEOA group also exhibited the highest titer, yield, and productivity among the TEOA-treated groups (Fig. 3D).

Notably, our results are in agreement with previous studies reporting that the pellet form of filamentous fungi is conducive to producing a higher product production [35, 36]. Moreover, we confirmed that the addition of 1.5% TEOA into the seed culture medium is conducive to efficient organic acid fermentation by *R. oryzae*. The increased biomass of the seed culture may lead to a significant improvement in the titers of fumaric and L-malic acid. Additionally, we also tested the fermentation parameters of *R. oryzae* seed pellets induced by low-pH (pH ~ 2.5) (Additional file 1: Fig. S3), which is currently a common strategy of inducing the pellet formation [37]. The results indicated that the productions of fumaric acid and L-malic acid were only 12.64 ± 0.24 and 4.02 ± 0.30 g/L, respectively, extremely demonstrating that our TEOA-induced seed pellet formation strategy were comparable to the traditional method (pH ~ 2.5).

### Transcriptome analysis of the possible mechanism driving pellet formation

**Fig. 4:**
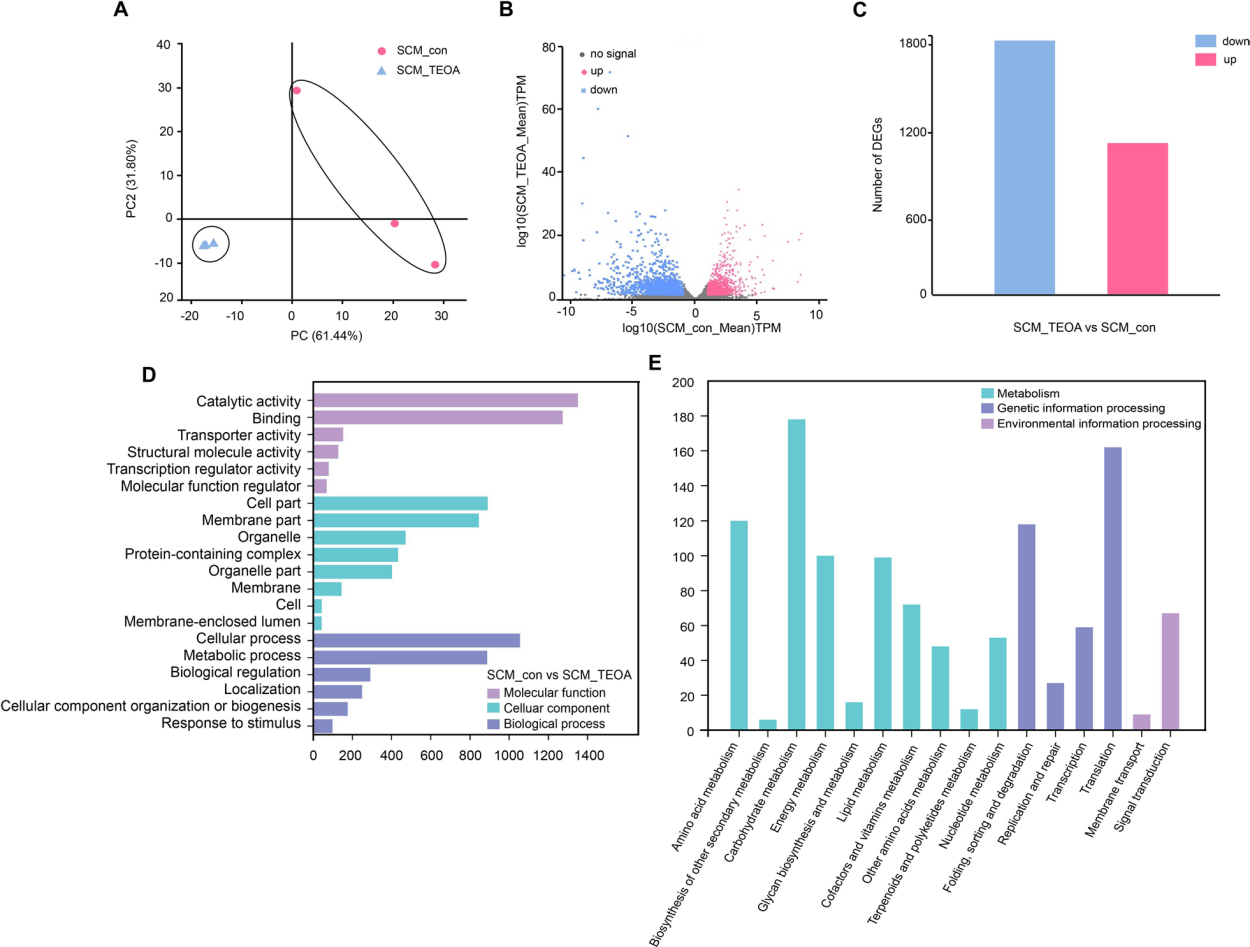
Transcriptomic profiling and annotation of DEGs between the seed culture medium with triethanolamine (SCM_TEOA)-treated group and the seed culture medium with surfactant-free (SCM_con) group. **A** score plots of the transcriptome of the SCM_TEOA group compared to the SCM_con group. **B** Volcano plots of the transcriptome of the SCM_TEOA group compared to the SCM_con group. **C** Up- and down-regulated DEGs in the SCM_TEOA compared to the SCM_con group. **D** GO annotation of DEGs in the SCM_TEOA group compared to the SCM_con group. **E** KEGG annotation of the DEGs in the SCM_TEOA group compared to the SCM_con group

In order to explore the molecular mechanisms underlying the morphological changes induced by TEOA, we performed RNAseq analysis to compare the transcriptome profiles of *R. oryzae* grown with or without TEOA during the seed culture process. We identified a total of 7783 common genes that were expressed in the cells treated with 1.5% TEOA and the untreated control group. We next conducted principal component analysis (PCA) of the transcriptome data and found that genes from the group grown in seed culture medium with TEOA (SCM_TEOA) and untreated control group (SCM_con) could be clustered into two distinct groups, showing that the transcriptional state of *R. oryzae* was significantly influenced by TEOA (Fig. 4A). Volcano plots were used to visualize the relative abundance of genes that were up- or down-regulated in the SCM_TEOA group compared to the SCM_con group (Fig. 4B). A total of 1094 differentially expressed genes (DEGs) were upregulated and 1841 downregulated (Fig. 4C, Additional file 2: Table S1 and Additional file 3: Table S2).

Subsequently, we conducted Gene Ontology (GO) annotation and classification analysis to reveal the potential functions of the DEGs that respond to TEOA treatment. The 2935 DEGs were categorized into 20 functional groups belonging to 3 domains, including molecular functions (6 groups), cellular components (8 groups), and biological processes (6 groups) (Fig. 4D). We found that catalytic activity, cellular component, and cellular process were the most abundant annotation terms in each of the three GO categories. Additionally, we also detected that a large of number of DEGs were related to the categories of metabolic process, membrane component, or binding (Fig. 4D). These findings confirmed that *R. oryzae* activated multiple complex pathways during the seed pellet formation process in response to TEOA. The results of our transcriptomic analysis were in agreement with the apparent morphological changes in *R. oryzae* seed pellets induced by the addition of TEOA. We speculated that these obvious changes resulted from differences in the expression levels of genes related to cellular components and processes.

**Fig. 5:**
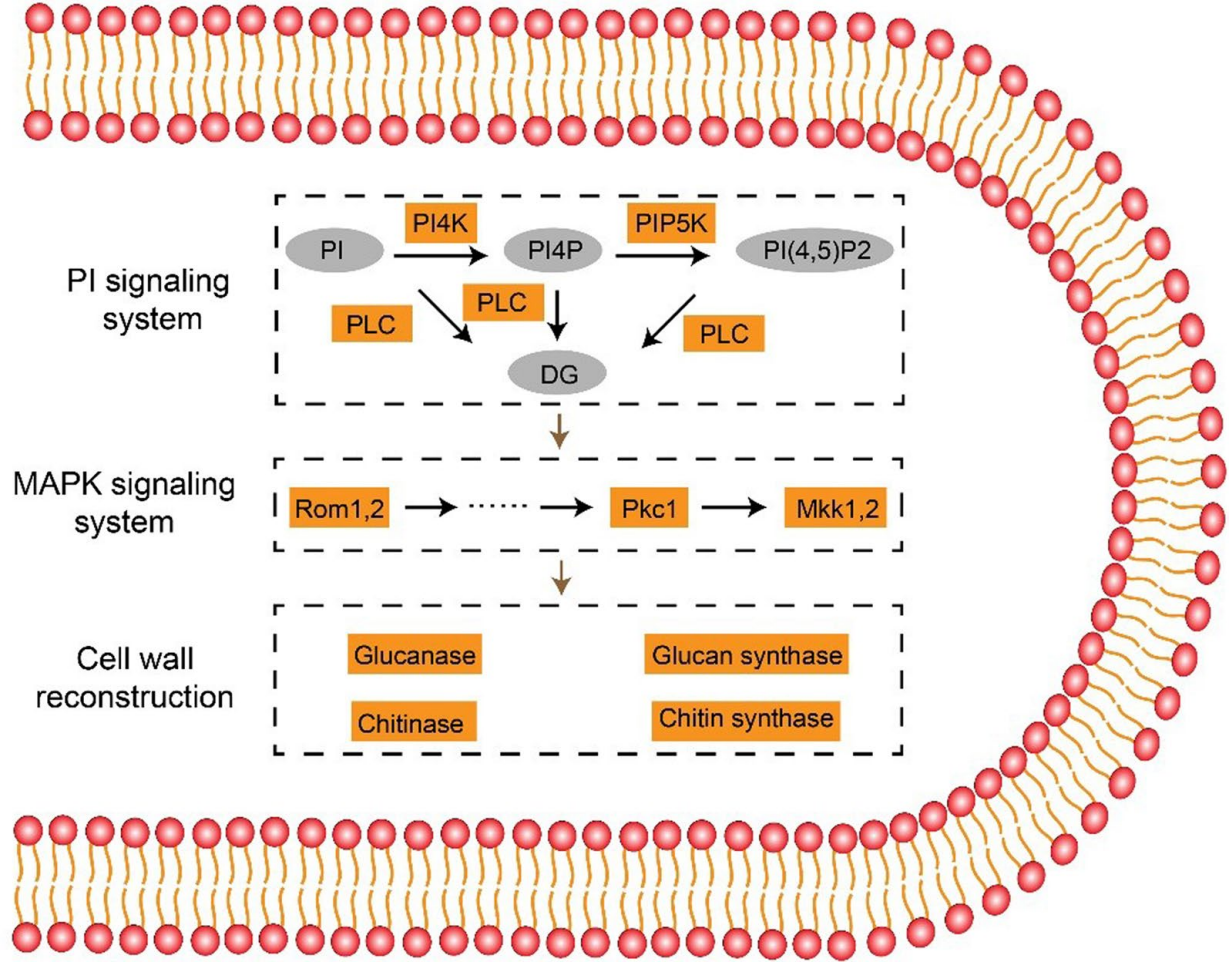
Proposed cell signal transduction pathway regulating cell wall reconstruction during TOEA-induced pellet formation

Additionally, we conducted Kyoto Encyclopedia of Genes and Genomes (KEGG) annotation. We observed moderately high numbers of DEGs related to carbohydrate metabolism, amino acid metabolism, and energy metabolism (Fig. 4E). This finding was complementary with our biomass data measured after the seed culture process, in which the growth of *R. oryzae* was suppressed. Thus, genes related to carbohydrate metabolism exhibited greatly different expression following TEOA treatment, resulting in a lower biomass. Given that diverse morphologies of *R. oryzae* seed cultures demonstrating distinctly different contents of cell wall and cell membrane components, the DEGs were partly concentrated in the category carbohydrate metabolism, lipid metabolism, and glycan biosynthesis. Moreover, we also observed that quite a large number of DEGs were enriched in genetic information processing, while several DEGs were concentrated in environmental information processing. Consequently, combined with previous studies on fungal morphology and related mechanisms, we inferred a framework of probable overall regulatory pathways consisting of three parts: an upstream signaling pathway, an intermediary signaling cascade, and a downstream cell wall reconstruction pathway (Fig. 5).

Morphological changes in *R. oryzae* include apical growth of hyphae and enhanced mycelial branching, which is accompanied by cell wall reconstruction [25, 38]. It should be noted that reorganization of cell wall architecture involves simultaneous activation of both degradation and formation processes in order to maintain the cell wall integrity [39]. In our transcriptome data, we found that DEGs that were significantly upregulated in the SCM_TEOA group were mainly involved in the phosphatidylinositol (PI) signaling system (11 up- vs. 5 down-regulated DEGs) and mitogen-activated protein kinase (MAPK) signaling (35 up vs. 18 down). These results were in agreement with previous reports that PI signaling and MAPK signaling play major roles in cell wall reconstruction [40]. We therefore focused on these two signal pathways to uncover the probable mechanisms driving pellet formation in response to TEOA treatment during the seed culture process.

Phosphoinositides (PIs) are short-lived membrane phospholipids with vital roles in various cellular functions including cytoskeleton regulation and motility, signaling, gating of ion channels, and the regulation of intracellular membrane traffic [41, 42]. In our study, KEGG analysis indicated that the upregulated DEGs in the SCM_TEOA group were significantly enriched in the PI signaling pathway, leading us to hypothesize that TEOA could directly or indirectly activate PI signaling. Then, PI is gradually converted by phosphatidylinositol 4-kinase (PI4K) (which was induced 1.4-fold over its expression in untreated cells), and phosphatidylinositol 4-phosphate 5-kinase (PIP5K) (1.7-fold greater expression than in untreated cells), to from phosphatidylinositol 4-phosphate (PI(4)P) and phosphatidylinositol (4,5)-diphosphate (PI(4,5)P2) (Fig. 5). Subsequently, phospholipase C (PLC), an essential catalytic enzyme in the PI signaling pathway, hydrolyzes PI, PI(4)P, and PI(4, 5)P2 into the secondary messenger diacyl glycerol (DG), which can subsequently activate members of the protein kinase C (pkC) family, thereby initiating a MAPK signal cascade [43] (Fig. 5).

The MAPK pathway plays a major role in signal transduction during the regulation of various physiological activities, such as cell wall integrity, stress response, and response to high osmolarity (HOG) [44–46]. Studies have shown that genes in the MAPK pathway can regulate the growth and metabolism of many fungal species, including *Coprinopsis cinerea*, *Tuber melanosporum*, *Neurospora crassa*, and *Pleurotus ostreatus* [47–49]. We observed that 35 up- and 18 down-regulated DEGs were enriched in the MAPK signaling pathway. In particular, we found that in the SCM_TEOA group, most of the up-regulated DEGs were associated with cell wall stress in MAPK sub-pathways, including Rom1,2, Pkc1, and Mkk1,2. Previous studies confirmed that Pkc1 and Rom1,2 could be activated by the PI signaling system, and our transcriptome analysis was consistent with these reports (3.3- and 3.1-fold greater expression than in untreated cells) [50, 51]. In addition, previous reports suggested that Mkk1,2 could be phosphorylated during the activation of the cell integrity pathway, and in our study, Mkk1,2 was significantly upregulated 4.8-fold [52]. Based on these results, it was reasonable to speculate that the cell wall stress-related MAPK sub-pathway was activated by TEOA treatment, ultimately leading to cell wall reconstruction and pellet formation.

MAPK pathway activation could further stimulate downstream enzymes required for the synthesis of components and cell wall reconstruction. Since carbohydrate-active enzymes (CAZymes) catalyze the cleavage, biosynthesis, and modification of complex carbohydrates crucial for cell wall remodeling and degradation, we also examined our transcriptomic dataset for significant differentially regulated CAZymes [53, 54]. We identified several up- and down-regulated CAZymes in the SCM_TEOA group, including glucanase, chitinase, glucan synthase, and chitin synthase. It is well known that the structure of fungal cell walls is highly dynamic, undergoing perpetual changes throughout the fungal life cycle [55]. Compared with the untreated control group, the *R. oryzae* SCM_TEOA group underwent obvious, substantive morphological changes, such as inhibited lengthening of the hyphal tip, suggesting that the *R. oryzae* cell wall is likely stretched and restructured during pellet formation. To affect this architectural shift, the main components of the fungal cell wall, i.e., chitin and glucan, must be at least partially hydrolyzed and resynthesized.

Our findings support the up- and down-regulation of these processes in response to TEOA exposure, which was consistent with previous reports showing that *β*-1,3-glucanase, *β*-1,6-glucanase, chitinase, and chitin synthase were associated with maintaining cell wall plasticity [56, 57]. These hydrolases and synthases may simultaneously act on the fungal cell wall, breaking and reforming bonds within and between polymers, eventually resulting in the reconstruction of the cell wall, as can be seen in *R. oryzae* pellets. Taken together, the data indicated that during the TEOA-treated seed culture process, the PI signaling system serves as a switch that actives the MAPK signaling pathway, ultimately resulting in cell wall reconstruction by regulating glucan and chitin metabolism (Fig. 5).

## Conclusions

In summary, we show that pellet formation in seed cultures of *R. oryzae* induced by exposure to TEOA can effectively promote higher organic acid production in subsequent fermentations, with 293 and 177% respective increases in the production of fumaric and L-malic acid. Transcriptomic analysis revealed that treatment with 1.5% TEOA lead to the upregulation of PI and MAPK signaling pathways, apparently stimulating downstream CAZymes that likely function in restructuring the cell wall architecture associated with pellet morphology. This approach offers a facile method to improve the organic acid production of *R. oryzae*, and offers a framework for new research efforts in manipulating the morphology of filamentous fungi to optimize their performance in various bioprocesses.

## Supplementary Information

**Additional file 1:** **Figure S1.** Representative digital images of *R. oryzae* seeds after seed culture medium supplementing with different surfactants (1.5% V/V). **Figure S2.** Assessment of *R. oryzae* colony cultured on different TEOA concentrations supplemented PDA agar medium. A, Digital images of *R. oryzae* morphology induced by different concentration of TEOA; B, Diameters of *R. oryzae* colony induced by different concentration of TEOA. Data represent means +/− SD of three independent replicates. Statistical significance was determined by Student’s t test (n= 3). * p < 0.05. **Figure S3.** Productions of organic acid after 72 h fermentation using low-pH induced *R. oryzae* seed pellets. Data represent means +/− SD of three independent replicates.

**Additional file 2**. Table S1. Upregulated DEGs.

**Additional file 3**. Table S2. Downregulated DEGs.

## Data Availability

All data generated or analyzed during this study are included in this manuscript and its Additional files.
