# Supplementary material for: Transcriptome analysis of Rhizopus oryzae seed pellet formation using triethanolamine
Source: Biotechnol Biofuels. 2021 Dec 4;14:230. doi: 10.1186/s13068-021-02081-y (PMC8645130; doi:10.1186/s13068-021-02081-y)
Supplement: Supplementary file 1 — Additional file 1: Figure S1. Representative digital images of R. oryzae seeds after seed culture medium supplementing with different surfactants (1.5% V/V). Figure S2. Assessment of R. oryzae colony cultured on different TEOA concentrations supplemented PDA agar medium. A, Digital images of R. oryzae morphology induced by different concentration of TEOA; B, Diameters of R. oryzae colony induced by different concentration of TEOA. Data represent means +/− SD of three independent replicates. Statistical significance was determined by Student’s t test (n= 3). * p < 0.05. Figure S3. Productions of organic acid after 72 h fermentation using low-pH induced R. oryzae seed pellets. Data represent means +/− SD of three independent replicates. [file 13068_2021_2081_MOESM1_ESM.docx]

**Supplementary material for**

**Transcriptome analysis of *Rhizopus oryzae* seed pellet formation using triethanolamine**

Na Wu^1^, Jiahui Zhang^1^, Wen Ou^1^, Yaru Chen^1^, Ru Wang^1^, Ke Li^1^, Xiao-man Sun^1^, Yingfeng Li^1^, Qing Xu^*1^, He Huang^*1^

1 School of Food Science and Pharmaceutical Engineering, Nanjing Normal University, Nanjing, China

*Corresponding author

E-mail: [xu_qing@njnu.edu.cn](mailto:xu_qing@njnu.edu.cn) and huangh@njnu.edu.cn


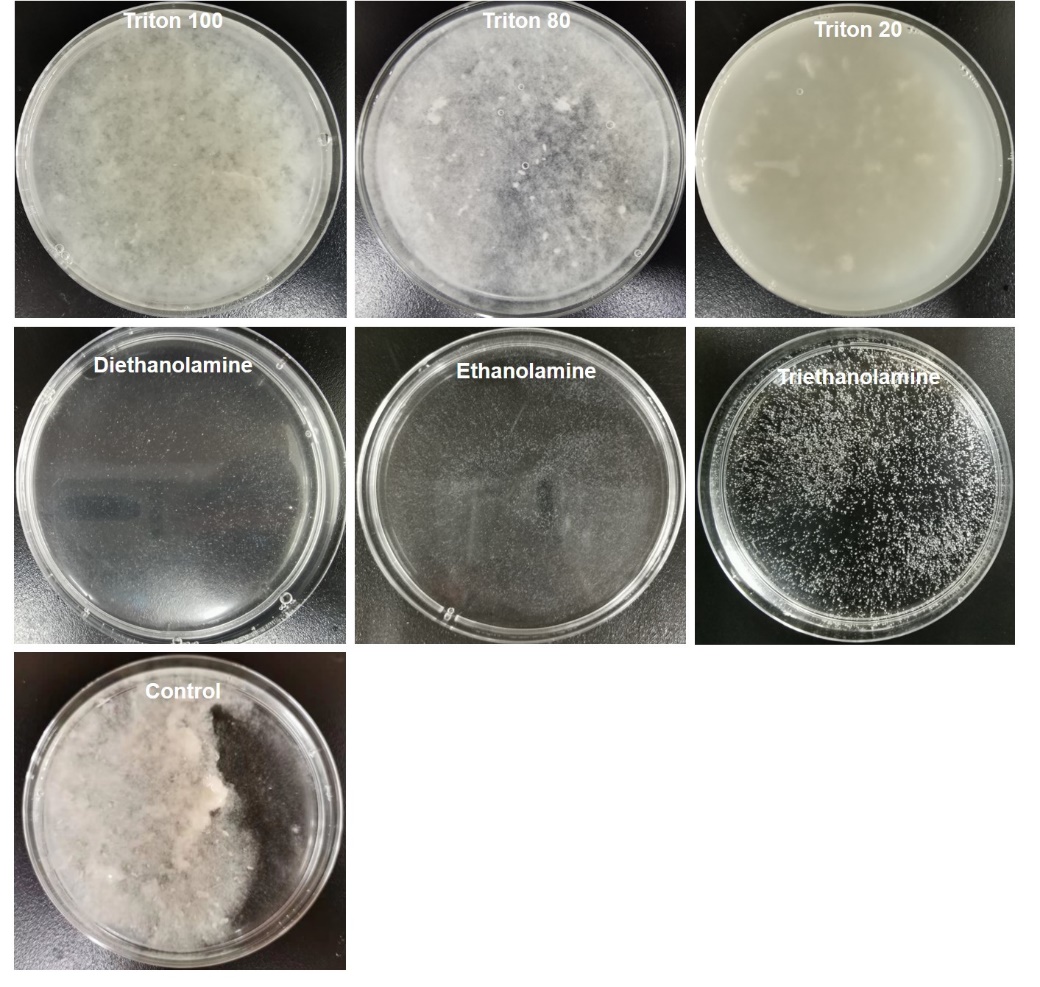


**Figure S1** Representative digital images of *R. oryzae* seeds after seed culture medium supplementing with different surfactants (1.5% V/V).


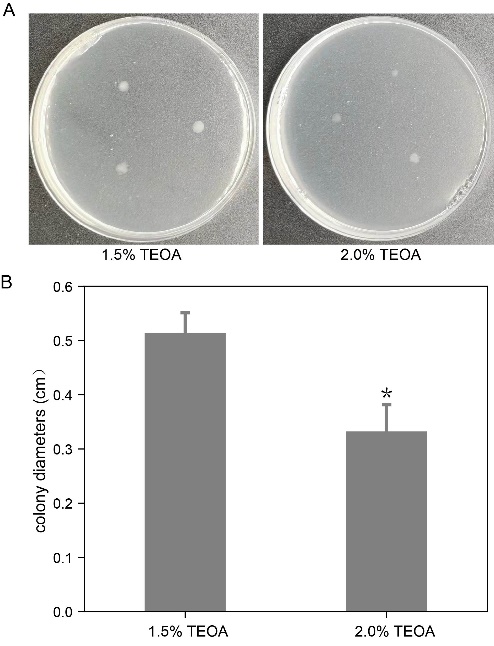


**Figure S2 Assessment of *R. oryzae* colony cultured on different TEOA concentrations supplemented PDA agar medium.** **A**, Digital images of *R. oryzae* morphology induced by different concentration of TEOA; **B**, Diameters of *R. oryzae* colony induced by different concentration of TEOA. Data represent means +/- SD of three independent replicates. Statistical significance was determined by Student’s t test (n= 3). ＊ p < 0.05.


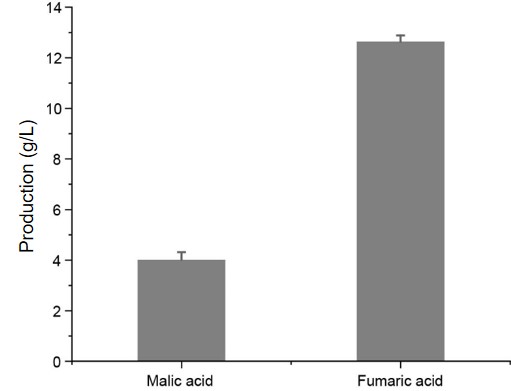


**Figure S3** Productions of organic acid after 72 h fermentation using low-pH induced *R. oryzae* seed pellets. Data represent means +/- SD of three independent replicates.
